# Cellular stress signaling and the unfolded protein response in retinal degeneration: mechanisms and therapeutic implications

**DOI:** 10.1186/s13024-022-00528-w

**Published:** 2022-03-28

**Authors:** Todd McLaughlin, Andy Medina, Jacob Perkins, Maria Yera, Joshua J. Wang, Sarah X. Zhang

**Affiliations:** 1 Department of Ophthalmology and Ira G. Ross Eye Institute, Jacobs School of Medicine and Biomedical Sciences, University at Buffalo, State University of New York, 955 Main Street, Buffalo, NY 14203 USA; 2 Neuroscience Program, Jacobs School of Medicine and Biomedical Sciences, University at Buffalo, State University of New York, Buffalo, NY USA; 3 Department of Biochemistry, Jacobs School of Medicine and Biomedical Sciences, University at Buffalo, State University of New York, Buffalo, NY USA

**Keywords:** Unfolded protein response, Metabolism, Endoplasmic reticulum stress, Retinal degeneration, Aging, Age related macular degeneration, Retinitis pigmentosa, Glaucoma, Diabetic retinopathy

## Abstract

**Background:**

The retina, as part of the central nervous system (CNS) with limited capacity for self-reparation and regeneration in mammals, is under cumulative environmental stress due to high-energy demands and rapid protein turnover. These stressors disrupt the cellular protein and metabolic homeostasis, which, if not alleviated, can lead to dysfunction and cell death of retinal neurons. One primary cellular stress response is the highly conserved unfolded protein response (UPR). The UPR acts through three main signaling pathways in an attempt to restore the protein homeostasis in the endoplasmic reticulum (ER) by various means, including but not limited to, reducing protein translation, increasing protein-folding capacity, and promoting misfolded protein degradation. Moreover, recent work has identified a novel function of the UPR in regulation of cellular metabolism and mitochondrial function, disturbance of which contributes to neuronal degeneration and dysfunction. The role of the UPR in retinal neurons during aging and under disease conditions in age-related macular degeneration (AMD), retinitis pigmentosa (RP), glaucoma, and diabetic retinopathy (DR) has been explored over the past two decades. Each of the disease conditions and their corresponding animal models provide distinct challenges and unique opportunities to gain a better understanding of the role of the UPR in the maintenance of retinal health and function.

**Method:**

We performed an extensive literature search on PubMed and Google Scholar using the following keywords: unfolded protein response, metabolism, ER stress, retinal degeneration, aging, age-related macular degeneration, retinitis pigmentosa, glaucoma, diabetic retinopathy.

**Results and conclusion:**

We summarize recent advances in understanding cellular stress response, in particular the UPR, in retinal diseases, highlighting the potential roles of UPR pathways in regulation of cellular metabolism and mitochondrial function in retinal neurons. Further, we provide perspective on the promise and challenges for targeting the UPR pathways as a new therapeutic approach in age- and disease-related retinal degeneration.

## Background

The retina is a thin layer of neural tissue that lies at the back of the eye and is responsible for sensing and processing the light input to generate visual signals and transmitting the information to the brain via the optic nerve. The vertebrate retina develops embryonically as an evagination from the developing neural tube and is thus part of the central nervous system (CNS) [1]. The structure of the retina is highly organized, consisting of multiple layers of photosensory neurons (photoreceptors), interneurons (bipolar cells, amacrine cells, and horizontal cells), projection neurons (retinal ganglion cells, RGCs), and their synapses. In addition, retinal blood vessels, which are enriched in the inner retina, and glial cells (astrocytes, Müller cells, and microglia) function as the supporting systems and form an integrated network with retinal neurons to maintain the metabolic and immune homeostasis in the retina. In mammals, retinal neurons are terminally differentiated at the early stage of life and do not regenerate [2]. Thus, severe injuries and loss of retinal neurons, such as light-sensing photoreceptors and projection neurons (RGCs), are often irreversible and subsequently lead to significant degeneration of the retina and catastrophic vision loss and blindness.

Relative to other CNS counterparts, retinal neurons are subjected to a greater level of environmental challenges and stresses [3, 4]. For example, retinal photoreceptors are constantly exposed to light, which can cause light toxicity and oxidative damage. In addition, photoreceptor cells have a high metabolic demand and a high protein turnover rate to maintain their physiological function and structural integrity [4]. The outer segments (OS) of photoreceptors, as the major site for visual phototransduction, are composed of highly specialized, disc-like structures enriched in lipids and proteins, which are prone to light-induced oxidative damage. To overcome the damage, the photoreceptor OS undergo daily shedding and renewal [5]. This process requires constant synthesis and proper folding of new proteins. In addition, major functions of photoreceptors, including phototransduction and neurotransmission, consume significant amounts of energy. These unique characteristics make photoreceptors highly susceptible to perturbations in the mitochondria and ER, which are the central hubs that govern metabolic and protein homeostasis.

To cope with the stress conditions, cells have developed a broad range of sophisticated stress response mechanisms to prevent and mitigate potential damages. These cellular signaling pathways, activated by distinct stressors, attempt to return the cell to homeostasis. However, if the stress conditions cannot be resolved, cells will activate programmed cell death signaling to eliminate damaged cells. Therefore, the stress response pathways are not only critical to maintaining long-term retinal integrity and function, but may also participate in disease pathophysiology by promoting cell death and degeneration. In addition to intrinsic stresses in retinal neurons, metabolic changes resulting from dysfunction and loss of retinal blood vessels, which reduces oxygen and nutrient supply to the retinal tissue, are also a frequent cause of neuronal death and degeneration. This can be seen in a number of ischemic retinal diseases such as diabetic retinopathy (DR) [6]. Imbalance of retinal microenvironment, governed by the blood-retinal barrier (BRB) consisting of tight junctions between neighboring vascular endothelial cells (inner BRB) or retinal pigment epithelium (RPE) (outer BRB), and glial cells, can activate cellular stress signaling in retinal neurons ultimately impacting their survival and function, resulting in vision impairment and blindness.

**Fig. 1:**
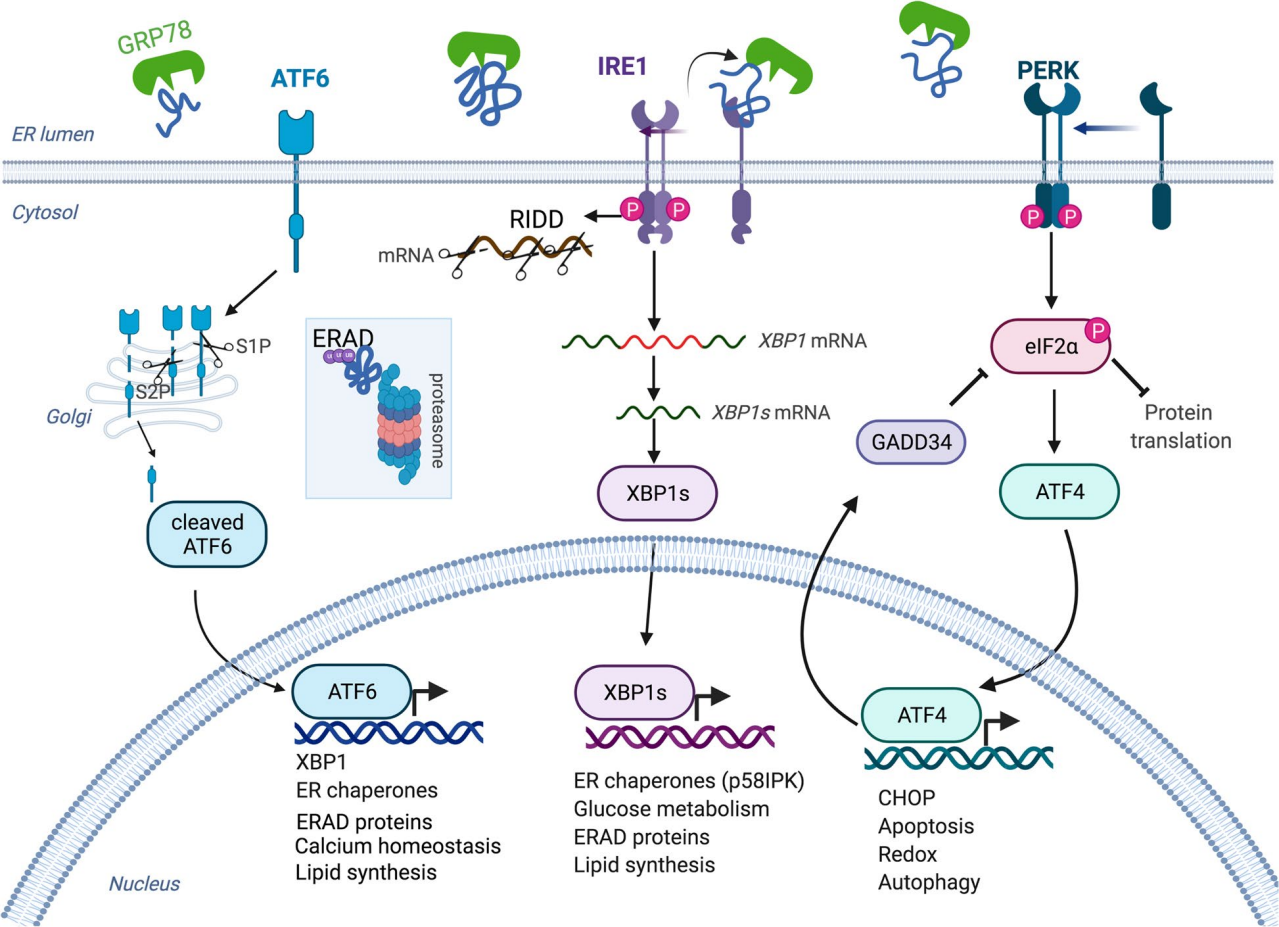
Schematic diagram depicts the three branches of the unfolded protein response (UPR) and resulting downstream targets reviewed in the context of aging and retinal disease. Each branch is activated when the resident ER chaperone, GRP78, releases from IRE1, PERK, and ATF6 to bind accumulated unfolded or misfolded proteins. The resultant signaling cascades activate downstream effectors, such as XBP1 and ATF4, in an attempt to re-establish homeostasis through multiple means including repressing protein translation, promoting ERAD (endoplasmic reticulum associated protein degradation) and RIDD (regulated IRE1-dependent decay of mRNA), and upregulating the expression of ER chaperones and foldases. In addition, the UPR pathways regulates genes involved in a broad range of ER stress-dependent and independent cellular processes including autophagy, glucose metabolism, lipid synthesis, cytoskeletal reorganization, and calcium regulation

Among the various types of cellular stress responses, ER-associated signaling pathways, including the unfolded protein response (UPR), ER-associated degradation (ERAD), autophagy, and integrated stress response (ISR), play a central role in promoting and maintaining a balanced and functional proteome in a cell. The UPR is activated upon a stress condition, where excessive unfolded or misfolded proteins accumulate in the ER, referred to as ER stress. To alleviate ER stress, the ER resident chaperone protein glucose-regulated protein 78 (GRP78; also known as immunoglobulin binding protein, BiP), dissociates from trans-ER membrane proteins activating transcription factor 6 (ATF6), inositol requiring enzyme 1 (IRE1), and PKR-like endoplasmic reticulum kinase (PERK). Subsequently, GRP78 binds to unfolded and misfolded proteins to promote their folding or refolding and as well keep them in a soluble form to prevent protein aggregation [7]. The dissociation of GRP78 from ATF6, IRE1, and PERK activates each of these proteins, which serve as ER stress sensors, and their downstream signaling cascades (Fig. 1). These signaling pathways work synergistically to restore the ER homeostasis via a variety of processes including increasing protein degradation, decreasing protein translation, and increasing production of chaperones and foldases that facilitate protein folding [7]. Activation of the UPR is an important mechanism required for cells to maintain the protein and ER homeostasis, especially in neural tissues such as the retina [8]. In addition, the UPR has been linked to a wide array of physiological processes such as glucose and lipid metabolism, mitochondrial function, redox regulation, calcium homeostasis, autophagy, just to name a few [9]. Understanding the role and regulation of the UPR in retinal development, maintenance, and aging, and its implication in retinal dysfunction and degeneration, could provide novel insights into the pathogenesis of retinal disease and lead to new treatments.

Here, we describe recent advances in understanding the mechanisms and signaling pathways of cellular stress response, with a major focus on the UPR, in retinal cells during aging and common retinal diseases, such as age-related macular degeneration (AMD), retinitis pigmentosa (RP), achromatopsia, glaucoma, and diabetic retinopathy (DR). We highlight a potential role of the UPR in regulation of cellular metabolism and mitochondrial function in retinal neurons and their therapeutic implications in protecting against age- and disease-related retinal degeneration and restoring neuronal and synaptic function.

### Aging

Aging is a major risk factor for chronic human disease, including a broad range of neurodegenerative diseases in the eye. Epidemiologic research demonstrates that the frequency of visual impairment from all causes increases significantly past the age of 60 and the prevalence of common retinal diseases such as AMD, DR, and glaucoma, also increases with age [10, 11]. Aging is a multifaceted process in which accumulation of stress over time results in alterations in cellular signaling, metabolic control, and protein homeostasis, ultimately causing substantial changes in morphology, structure, and function in cells and tissues. Clinical studies have shown a continuous decline of retinal function with aging in normal human subjects aged 10 to 69 years and a reduction in central retinal thickness and retinal nerve fiber layer thickness in elderly population with age of 65 years or older [12, 13]. In experimental models, wild-type mice after 12 months of age demonstrate decreased retinal thickness, reduced retinal function, and a loss of retinal neurons including RGCs, bipolar cells, and peripheral photoreceptors [14–16]. In addition, the dendritic field size in subtypes of RGCs decreases with aging, suggesting that morphological changes other than cell loss of retinal neurons also contribute to age-related functional deficits [17]. Loss of synapses and increased synaptic remodeling in the neural retina is another characteristic of aged retina, which is evidenced by fewer photoreceptor synapses and displaced presynaptic photoreceptor ribbons from the outer plexiform layer (OPL) to the outer nuclear layer (ONL) along with aberrantly extended bipolar dendrites in mouse models of premature aging [18–21]. Interestingly, retraction of photoreceptor synapses has also been reported in human retinal degenerative diseases, such as AMD, retinitis pigmentosa, and retinal detachment [22]. These overlapping phenotypes suggest common underlying mechanisms for retinal degeneration during aging and disease conditions.

A functional UPR for maintaining the protein and ER homeostasis is critical for healthy aging [23]. As the organism ages, the expression levels of UPR proteins show changes and the ability of the cell to respond to cellular stress declines. In human lens, the baseline levels of GRP78, IRE1, and ATF6 increase progressively from ages 50 to 90 years [24]. Similarly, the levels of C/EBP homologous protein (CHOP) increase in aged mouse brain and retina [25]. Conversely, the baseline level of spliced XBP1 (XBP1s; the activated form of XBP1) decreases with age in the mouse retina [18]. In human, this variation extends to the individual with aged monozygotic twins showing differential expression of XBP1s correlated to cognitive function [26]. Furthermore, the changes in UPR components appear to be tissue-specific. For example, phosphorylated PERK levels are reduced in aged pancreas but increased in aged kidney [27, 28]. In aged rat retina, effectors in the PERK pathway, such as phosphorylated eukaryotic translation initiation factor-2α (eIF2α) and NF-E2-related factor 2 (Nrf2) are reduced, whereas other downstream effectors, such as ATF4 and CHOP, are elevated compared to younger controls [29]. These changes may suggest an increase in cellular stress in the ER coupled with disrupted protein homeostasis. In human retina, the presence of protein aggregates of nonphosphoylated tau and α-synuclein increases substantially with advanced age, further supporting the presence of protein misfolding and dyshomeostasis in aged retinas [30].

In addition to the changes in the basal levels of UPR proteins, the ability of aging cells to respond to cellular stress declines [31, 32]. Our recent study has shown that the ER stress stimulator, thapsigargin, was able to induce a robust activation of the UPR in the retina of young adult mice but failed to increase XBP1s expression in the retina of 13-month-old mice [18]. This finding suggests that declined function of the UPR pathways may contribute to neuronal dysfunction and degeneration in aging mice [18] and retinal diseases [33]. Further supporting this notion, conditional knockout (cKO) of XBP1 in retinal neurons results in accelerated retinal degeneration and retinal function decline with aging. At the age of 12–14 months, XBP1 cKO mice show significant structural and functional deficits that resemble wild-type mice twice that age, including reduced retinal thickness, loss of RGCs, and morphological defects of retinal synapses [18, 20]. Remarkably, a strikingly similar phenotype featuring age-related increase in ectopic photoreceptor-bipolar synapses is also observed in ER membrane protein complex 3 (Emc3) cKO mice [21], liver kinase B1 (Lkb1) cKO mice, and AMP activated protein kinase, alpha 1 and 2 subunits (AMPKα1/AMPKα2) double cKO mice [19]. Functionally, both light- and dark-adapted electroretinograms (ERG) show reduced amplitudes in all of these aging cKO models; the optokinetic response also deteriorates in mice with aging [15, 18, 20, 21]. AMPK functions as an energy sensor, whose activation increases glucose uptake and glycolysis, promotes fatty acid oxidation, and enhances mitochondrial biogenesis to restore energy supply and balance [34]. These findings suggest that maintaining the ER homeostasis and energy metabolism is critical for retinal neuronal survival and function during aging. Intriguingly, the retinas from aged XBP1 cKO mice have an overall decrease in baseline glycolysis and in maximum glycolytic response, compared to age-matched wild-type mice, and these changes may contribute to accelerated retinal neurodegeneration in these mice [12]. A progressive decline in metabolic control due to impaired function of nutrient-sensing pathways results in perturbations in energy metabolism in aged animals [35]. Together, these studies suggest restoring the UPR function may protect against metabolic defects, thus reducing the long-term stress associated with aging and tissue deterioration in age-related disease.

### Age-related macular degeneration

Age-related macular degeneration (AMD) is a leading cause of severe, irreversible vision loss in elderly populations [36]. Approximately 10% of individuals over the age of 65 years and 25% of those over the age of 75 years in developed countries have been diagnosed with AMD. As life expectancy increases, so too does the prevalence of AMD. It is expected that by 2040, nearly 300 million people worldwide will be affected by the disease [37, 38]. The rapid increase in disease prevalence renders AMD a significant global health concern that negatively influences the well-being of the population. Clinically, AMD can be categorized into two stages, early and late AMD. Early stages of the disease are characterized by small extracellular deposits or drusen, depigmentation of the retinal pigment epithelium (RPE) layer, and impaired RPE functionality [39, 40]. Advanced stages of the disease can be subclassified into non-neovascular (or dry) and neovascular (or wet) AMD. Both forms of advanced-stage AMD are accompanied by loss of photoreceptors and geographic atrophy (GA), but neovascular AMD (nAMD) is distinguished by presence of pathological angiogenesis in the macula, or macular neovascularization (MNV) [41, 42]. According to the anatomic location and origination of the new vessels, MNV can be classified into three major types. Type 1 and Type 2 MNV originate from the choroid and proliferate under the RPE (Type 1) or breaks through the RPE to reach subretinal space (Type 2), while Type 3 MNV originates from the retina and grows toward the RPE [41]. Regardless of the type of the MNV, these malformed vessels lack appropriate pericyte coverage and tight junctions between endothelial cells and are therefore prone to leakage or rupture. Common lesions caused by MNV include exudation, hemorrhages, and edema in the macula, which is often associated with severe visual impairment [39, 43].

AMD is a multifactorial disease involving the interplay between advanced age, environmental risk factors, and genetic factors. Common variants found in the complement factor H (*CFH*) and age-related maculopathy susceptibility 2 (*ARMS2*) genes have been shown to increase the risk of AMD [39]. Environmental factors that are responsible in part for disease onset and progression include modifiable risk factors like cigarette smoke and diet, but also hyperopia, hypertension, and sex (female) [44, 45]. The complex etiology poses significant challenges to the development of therapeutics for AMD. Current standard treatment options include intravitreal anti-vascular endothelial growth factor (VEGF) therapies for MNV in patients with wet AMD or nAMD, which significantly reduce vascular leakage in most cases, and inhibit vascular growth in some; however, its overall long-term effect on MNV regression or inhibition of MNV expansion remains suboptimal [43, 46]. In addition, no effective treatment is available for patients with early AMD and late stage AMD with GA [47]. Limitations on treatment options for AMD leave much to be discovered regarding the pathophysiology of the disease and the underlying molecular mechanisms, particularly initiation of the early-stage damage and dysfunction of the RPE.

The RPE is a monolayer of cuboidal epithelial cells located between choroidal vasculature and the outer segments of the photoreceptors. Basolaterally, RPE cells form the outer BRB by tight junctions and adhere to a highly organized basement membrane, known as Bruch’s membrane, which separates RPE cells from fenestrated endothelium of the choroidal capillaries [48, 49]. Apically, the RPE faces the light-sensitive photoreceptor outer segments (POS) and plays a crucial role in nourishing the outer retina, detoxifying and phagocytosing damaged POS, and regenerating visual pigment to maintain the process of phototransduction. In addition, the RPE serves as an essential component of a metabolic ecosystem in the eye [50–52]. In this system, glucose from the choroid is transported through the RPE to photoreceptors; photoreceptors then convert glucose to lactate, which is provided as a fuel to the RPE and neighboring retinal cells [53]. Lactate also suppresses glycolysis in the RPE that further preserves glucose for use by photoreceptors [54]. Without an intact RPE, critical processes such as photoreceptor morphogenesis and metabolic homeostasis are impaired and photoreceptor cells are likely to undergo degeneration [55, 56].

A prominent characteristic of early AMD is the accumulation of drusenoid deposits in the subretinal space and the thickening of the Bruch’s membrane [39]. Possible contributing factors to these pathological changes include malfunction of macrophages that fail to remove cell debris from subretinal space [57], dysregulation of lipid metabolism associated with aging [58], and accumulation of lipoproteins in Bruch’s membrane [59]. In addition, disturbed protein homeostasis plays a central role in this process. In human donor eyes, accumulation of amyloid β, a major component of amyloid plaques found in the brains of the patients with Alzheimer’s disease, was observed in drusen, correlating with complement activation and RPE/photoreceptor degeneration in AMD [60–63]. Ubiquitin-positive aggregates were also identified in soft and hard drusen in aged human retinas [30]. These findings suggest an implication of protein dyshomeostasis in the pathogenesis of AMD. In parallel with drusen formation, accumulation of lipids and protein modifications in the extracellular matrix leads to structural and compositional changes in Bruch’s membrane (reviewed in [64]). These changes impair the bidirectional nutrient transfer from the RPE to the choriocapillaris, further contributing to RPE and photoreceptor degeneration.

In response to nutrient shortage and disturbed metabolism, cells activate adaptive signaling pathways and molecules, among which is the AMPK/mammalian target of rapamycin (mTOR) pathway [65]. Activation of AMPK increases energy production and regulates a wide variety of metabolism-related stress responses, such as anti-oxidant defense, autophagy and mitophagy [66]. In the RPE from human donor eyes with AMD, AMPK activity was drastically reduced, suggesting that insufficient AMPK activation may be implicated in AMD [65]. Pharmacological activation of AMPK by metformin (1,1-dimethylbiguanide hydrochloride) protects photoreceptors and the RPE from light- and oxidative stress-induced damage [67]; conversely, retina-specific knockout of AMPK leads to retinal dysfunction and age-related neurodegeneration, suggesting an essential role of AMPK in retinal neuronal survival and function [68]. Interestingly, conditional deletion of AMPK in the neuroretina also induces a secondary degeneration of the RPE, which is perhaps not surprising given the close interdependence between the RPE and the retina as a metabolic ecosystem. In contrast, in the context of glaucoma (discussed below), hyperactivation of AMPK results in significant morphological changes and functional decline in RGCs, whereas depletion of AMPK rescues both structure and function in RGCs [69]. These discrepant results suggest that AMPK may activate distinct downstream pathways that exert varying or even opposite effects on cell metabolism and stress response in different cell types (i.e. RPE cells and RGCs). Therefore, understanding cell-specific signaling pathways in response to distinct stressors is critical to the formulation of effective interventions.

Closely related to dysregulation of cellular metabolism are increased oxidative stress and ER stress, which play a major role in RPE damage and AMD pathogenesis [39]. Recent work highlights a close interplay between these two types of stress [58, 70]. Cigarette smoke, a major environmental risk factor, activates oxidative stress and ER stress in RPE cells resulting in RPE apoptosis and cell death, disruption of the barrier function, and thickening and deposit accumulation on Bruch’s membrane [71–75]. Inhibition of ER stress or reduction of oxidative stress both protect RPE cells from cigarette smoke extract (CSE)-induced apoptosis and cell death [74, 76]. Moreover, alleviating ER stress significantly reduces mitochondrial fragmentation and decreases reactive oxygen species (ROS) generation in CSE-challenged RPE cells, further suggesting a close interplay between ER stress and oxidative stress [76]. Increased oxidative stress stimulates an upregulation of genes, such as transcription factor, Nrf2, to restore redox homeostasis [76]. In aging RPE, the Nrf2 signaling was found less functional in response to oxidative stress, which makes aging RPE vulnerable to oxidative damage [77]. Overexpression of Nrf2 significantly improves survival and barrier function of RPE cells challenged with oxidative stress and in animal models of retinal degeneration [78]. Genetic and/or pharmacological approaches to enhance Nrf2 function hold great promise for developing new treatments for AMD and other retinal degenerative diseases.

Like oxidative stress, ER stress has been implicated in the RPE pathologies associated with AMD [3, 74, 76, 79, 80]. In response to ER stress induced by CSE, all three UPR branches can be activated [76]. Among these branches, the IRE1/XBP1 pathway has been shown to be essential for RPE survival and function during stress conditions and for maintaining the RPE structural integrity by regulating calcium-dependent RhoA/Rho kinase signaling and actin cytoskeleton organization [74, 79, 80]. In human RPE cells, inhibition of XBP1 intensifies CSE-induced apoptosis; in contrast, suppression of the PERK/ATF4/CHOP pathway improves RPE cell survival, suggesting that the XBP1 pathway and the PERK/ATF4/CHOP pathway play differential roles in RPE survival during AMD [74]. Interestingly, despite the pro-apoptotic role of CHOP in mediating ER stress-related cell death in many cell types, silencing of CHOP gene in the RPE results in reduced Nrf2 activation and a marked increase in apoptosis [76]. Similarly, deficiency of CHOP advances rod photoreceptor cell death in degenerative retinal diseases such as Retinitis Pigmentosa [81]. These results suggest that maintaining a certain level of CHOP is necessary for Nrf2 activation and cell survival in the RPE and photoreceptors during stress conditions. However, excessive CHOP activation by ER stress can be detrimental to cell survival and function contributing to neurodegeneration [82].

Past studies have highlighted the importance of molecular chaperone proteins in protecting the RPE during AMD pathogenesis. Endoplasmic reticulum protein 29 (ERp29) is a multifunctional ER chaperone belonging to the protein disulfide isomerase family. As a putative ER chaperone, ERp29 facilitates the folding and trafficking of secretory and membrane proteins, such as connexin 43, which is an integral membrane protein that forms the gap junctions [83]. In addition, ERp29 functions as a regulator of cellular stress response by direct interacting with PERK and ATF6 in the UPR pathways and upregulating/enhancing the function of other ER chaperones (reviewed in [84]). While highly expressed in normal secretory epithelial cells, the levels of ERp29 were found significantly reduced in the RPE in both AMD patients and cells exposed in vitro to CSE. Overexpression of ERp29 protected RPE cells from CSE-induced ER stress, tight junction damage, and apoptosis. In contrast, ERp29 knockdown leads to decreased activation of the ATF6 pathway, reduced levels of p58^IPK^ and Nrf2, and increased p-eIF2a and CHOP activation resulting in exacerbated CSE-triggered cell death [84–86]. Future studies are warranted to investigate the therapeutic potential of targeting specific protective UPR pathways, such as XBP1, or associated molecular chaperone proteins, such as Erp29, to restore the ER and protein homeostasis, for preventing RPE and photoreceptor damage in animal models of AMD.

### Retinitis Pigmentosa

Retinitis Pigmentosa (RP) represents a group of rare genetic diseases where mostly rod-specific gene mutations cause slow and progressive rod, and subsequently secondary cone, degeneration leading to vision loss [87]. Genetic mutations in over 50 causal genes of RP have been identified [88]. In part due to the diversity and relative rarity of each mutated gene, currently there is only one Food and Drug Administration-approved treatment for RP, specific to the RPE65 mutation [89]. RPE65 encodes an all-trans retinyl ester isomerase in the RPE essential for production of the photopigment 11-cis-retinal. More common forms of RP are associated with misfolding of proteins caused by mutations of the rhodopsin gene (RHO). The resultant rhodopsin protein is a seven-transmembrane G-protein-coupled receptor responsible for initiating the phototransduction cascade in rod photoreceptor cells [88, 90, 91]. Over 200 mutations of the RHO gene have now been identified and may be inherited in an autosomal dominant or less frequently in an autosomal recessive manner [92, 93]. Autosomal recessive RP (arRP) is characterized by homozygous recessive inheritance of loss-of-function RHO mutations, such as those found in Receptor Expression Enhancer Protein 6 (REEP6). Mutant REEP6 proteins lead to retinal degeneration through defective formation and localization of guanyl cyclases and consequent alteration of the phototransduction pathway [94–96]. More commonly implicated, autosomal dominant RP (adRP) mutations such as P23H (proline substituted by histidine at position 23) and T17M (threonine substituted by methionine at position 17) are thought to be responsible for 20–30% of all adRP cases [91, 92]. These mutations have been shown to increase ER stress and activate the UPR and ERAD pathways in photoreceptors [97]. Selective activation of IRE1 decreases misfolded rhodopsin proteins in both the P23H and T17M models as well as a non-class II mutant rhodopsin, S334ter rhodopsin, in part through degradation by both ERAD and regulated IRE1-dependent mRNA decay (RIDD) [98]. Recent studies have shown that robust rhodopsin degradation precedes retinal degeneration and the IRE1 signal transduction pathway remains activated even after photoreceptor degeneration plateaus [33, 99]. Further evidence of the beneficial role of IRE1 points to the molecular chaperone, ER degradation-enhancing a-mannosidase-like 1 (EDEM1), which assists in regulation of protein degradation in the ER [100–102]. As a component of the IRE1 pathway, EDEM1 accelerates degradation and clearance of P23H rhodopsin proteins and in doing so may also promote the proper folding and transport of folding-competent mutant proteins [102]. This suggests that photoreceptor death may not be associated with insufficient activation of the IRE1 pathway and other pathways may contribute to the degeneration process. However, the cytoprotective features of the IRE1 pathway, such as EDEM1’s dual role of enhancing mutant rhodopsin degradation and promoting folding-competent protein, may prove useful in therapeutic interventions aiming to alleviate protein misfolding [102, 103].

In contrast to the IRE1 pathway that promotes protein folding and ERAD to alleviate ER stress, activation of PERK increases the phosphorylation of eIF2α, resulting in a decrease in global protein synthesis and an increase in ATF4 production [98]. The role of the PERK/ATF4 pathway in the pathogenesis of RP has been studied by several groups [104, 105]. Inhibition of PERK with GSK2606414A increases the production of both normal and mutant rhodopsin proteins resulting in increased protein aggregation, reduced photoreceptor survival, and decreased visual function. In contrast, enhancing eIF2α phosphorylation protects photoreceptors in P23H rats, suggesting that PERK activation to reduce global protein synthesis thus alleviating protein aggregation and ER stress is likely a protective response at the early stage of the disease [105]. Despite the early activation of PERK protecting photoreceptors again proteotoxicity and ER stress, long-term activation of PERK induces an increase in ATF translation and an upregulation of its downstream effector CHOP [106]. In T17M RP mouse model, elevated ATF4 levels accompanied by increased CHOP expression and reduced autophagy contribute to photoreceptor degeneration in RP [104]. Intriguingly, ablation of CHOP showed no effect on reducing photoreceptor death in two RP models [81, 107]. The mechanism behind these observations is not well understood, but earlier studies revealed that deletion of CHOP reduces protein expression of Nrf2, a key protective factor against oxidative damage, in the RPE [76]. Investigation of the downstream targets of CHOP in photoreceptors may provide new insights into the role of CHOP in RP. In addition, the protective effects of the PERK pathways are likely necessary for long-term photoreceptor survival and visual function in adRP by reducing mutant rhodopsin retention in the ER and diminishing rod photoreceptor degeneration [33]. This duality of the PERK signaling pathway may be specific to adRP models, wherein ER stress induced by protein misfolding can be alleviated by reduction of overall protein synthesis and upregulation of molecular chaperones [108–111].

In response to rhodopsin misfolding and ER stress in photoreceptor cells of adRP, a third UPR pathway, mediated by ATF6, is also activated [112]. Activation of ATF6 upregulates ER chaperones, such as GRP78, to promote protein folding and restore ER homeostasis [113] [99, 111]. In addition, selective activation of ATF6 provides a protective action that can be closely tied to processes ensuring proper ER folding, such as ERAD. Independent of IRE1 and PERK, selective activation of ATF6 upregulated HMG-CoA reductase degradation protein 1 (HRD1) – dependent ERAD of amyloid precursor protein [114]. In adRP models, activation of ATF6 decreased the levels of class II mutant rhodopsin, including P23H and T17M, while sparing monomeric WT rhodopsin production [98]. As with the IRE1 pathway, elucidating the role of molecular chaperones involved in specific UPR branches may improve targeted gene therapies for adRP. Recent findings demonstrated that intravitreal AAV injection of the GRP78 chaperone alleviates ER stress, suppresses apoptosis, and improves ERG responses in a rat P23H RHO model [114]. GRP78 alongside the co-chaperone and ER DNAJ protein 5 (ERdj5/DNAJC10) are also required for formation of the C110-C187 disulfide bond in WT rhodopsin. Knockdown of ERjd5 decreased expression of WT and mutant P23H rhodopsin, suggesting the importance of DNAJ proteins in maintaining the ER stress response [110, 115]. Viral-mediated overexpression of GRP78 and ERdj5 further supports these findings with results showing an overall reduction in ER stress and enhanced photoreceptor cell survival in the P23H RHO mouse model [110, 114, 116]. Although ATF6 signaling ensures degradation of mutant rhodopsin proteins present in RP, it cannot regulate proper folding of mutant rhodopsin [111]. This contrasts with ER chaperones downstream of IRE1, like EDEM1, which possess both improved mutant rhodopsin degradation and restoration of folding-competent P23H rhodopsin [102]. Altogether, these recent findings elucidating the proposed mechanism of each UPR pathway presents new opportunities for targeted therapies focusing on individual branches of the UPR and their co-chaperones [98, 111, 114].

### Achromatopsia

Achromatopsia is a rare autosomal recessive disorder characterized by impaired cone photoreceptor function, leading to decreased visual acuity beginning at birth or early infancy, nystagmus, and reduced or absent color vision [117–119]. Six genes have been identified in close association with achromatopsia, including the gene encoding ATF6. Mutations of ATF6 result in autosomal recessive retinal cone dystrophy and convey increased susceptibility to ER stress from hypoxia, protein misfolding, and light damage [120–122]. In animal models, global ATF6 knockout mice show normal retinal morphology and function at a young age but develop photoreceptor dysfunction with increasing age [117]. Knockout of ATF6 in a P23H-KI model of RP impairs rhodopsin clearance and accelerates retinal degeneration and functional deficits [112]. A phenotypic correlation is seen in patients with ATF6 mutation-induced achromatopsia who present foveal hypoplasia, supporting a role of ATF6 in cone development [117, 121, 123]. Interestingly, using human stem cell-derived retinal organoids, a recent study shows that genetic variants that disrupt ATF6 function lead to impaired cone development and a loss of cone OS/IS [120]. The contradictory results from human and animal studies are believed to be associated with the intrinsic biologic differences and environmental factors that influence the role of ER stress and the UPR pathways in murine and human retinal development [117, 120, 123]. In addition, further insight into the presence of non-functioning peripheral cones may offer advances in pre-existing therapeutic interventions, such as gene therapy for achromatopsia associated with GNAT2, CNGA3, and CNGB3 mutations [117, 124, 125].

Recent investigations into the associations between ATF6, photoreceptor integrity, and achromatopsia reveal the diversity among the roles and potential mutations of ATF6. Current studies have begun to highlight these diverse molecular defects and the associated defects seen in specific steps of ATF6 activation. For example, Class 1 ATF6 mutants possess impaired trafficking from the ER to the Golgi apparatus whereas Class 3 mutations show an impaired basic leucine zipper (bZIP) domain [126]. Further exploration into the stepwise activation of ATF6 may prove of use for potential therapeutic strategies, including gene replacement therapy for defective transcriptional activators and gene editing for mononucleotide mutations. ATF6 small molecule agonists, such as ATF6-activating (AA) compounds AA147 and AA263, and antagonists, such as Ceapin-A7, have been shown to selectively modulate the ATF6 arm of the UPR pathway [108, 127, 128], Downstream targets of ATF6 may also serve as potential targets in achromatopsia. As seen in adRP models, overexpression of GRP78 and ERdj5 by AAV mediated delivery decreases aggregation of mutant proteins and may be possible regulators of ATF6 translocation to the nucleus [114, 116]. Although ATF6 is essential for regulating ER stress in retinal photoreceptors, the mechanisms behind ATF6-associated achromatopsia and its preference for central cone photoreceptor degeneration remains unclear. Future therapeutic interventions for achromatopsia, or any other AT6-associated disease conditions, must take into account that modulating ATF6 activation in cones may have catastrophic consequences for color vision. Thus, strategies targeting individual cell types (e.g. through specific viral variants) or specific regions (e.g. outer retina) should be considered over broad or systemic treatments.

### Glaucoma

Glaucoma is a leading cause of irreversible blindness characterized by progressive degeneration of RGCs and their axons resulting in a loss of visual field and central vision, if left untreated. In 2013, approximately 64.3 million people aged 40–80 years worldwide were affected by primary open-angle glaucoma (POAG) and primary angle-closure glaucoma (PACG) and the numbers were estimated to increase to 76.0 million in 2020 and 111.8 million in 2040 [129]. Vision loss in glaucoma often starts from the periphery and progresses without noticeable symptoms in patients until late stages. Thus, RGCs undergo a prolonged course of degeneration after the disease onset, which provides a valuable window for intervention upon a timely diagnosis. Glaucoma is multifactorial disease. Among many identified risk factors, elevated intraocular pressure (IOP) is the most predominant, and the only modifiable factor causing RGC degeneration. An increase in the IOP occurs as a result of a buildup of aqueous humor due to reduced drainage of aqueous fluid caused by a stiff and less permeable trabecular meshwork (TM) and increased outflow resistance at the TM [130, 131]. In addition to increased stiffness of the TM, there is also morphological and biochemical changes including extracellular deposits within the cribriform layer of the TM [132]. Current clinical treatment for glaucoma focuses on pharmacological, laser, or surgical therapies to lower IOP, either by increasing aqueous humor drainage or decreasing its production [133]. Experimentally, multiple mouse models have been developed to recapitulate increased IOP using a variety of techniques including intracameral injection of microbeads, laser photocoagulation, episcleral vein cauterization, and injection of hypertonic saline and hyaluronic acid [134]. Increased IOP leads to loss of RGCs and their axons and optic-disc cupping, suggesting a causal role of high IOP in glaucomatous RGC damage and neuropathy [134].

Genetic factors play an important role in the pathogenesis of glaucoma. Recent studies have identified multiple genomic loci and genetic variants that contribute to glaucoma development [135–137]. Paired Box Gene 6 (PAX6) is a transcription factor that regulates development of the eye and its dysregulation or mutation can lead to aniridia (a complete or partial absence of the iris) and congenital glaucoma [136, 138]. CAV1/CAV2 are genes that encode caveolin-1 and caveolin-2 proteins, respectively, which can bind to cholesterol and are therefore important in maintaining membrane homeostasis and cholesterol metabolism, as well as regulating TM outflow [135, 139]. GAS7 encodes growth arrest-specific protein 7, which plays a pivotal role in cell division and neuronal development [135, 137, 140]. These findings not only provide insights into the molecular mechanisms of glaucoma but also present an opportunity for developing genetic screening for early diagnosis and potentially for gene therapy or overexpression of functional proteins in RGCs.

#### Cell stress signaling in TM cell damage and increased IOP

Multiple studies have shown that dysregulation of the UPR pathways in TM cells are involved in the development of glaucoma. It is important to understand the mechanisms that lead to ER stress in TM cells in order to prevent the subsequent damage. Mutations in the MYOC gene, which encodes myocilin protein, have been linked to increased IOP in juvenile open-angle glaucoma (JOAG) and adult-onset POAG [141]. Mechanistically, mutations of myocilin cause protein misfolding resulting in accumulation of misfolded myocilin proteins in the ER and increased ER stress in TM cells [142, 143]. Treatment with phenylbutyric acid (PBA), a chemical chaperone that promotes protein folding and alleviates protein aggregation thus reducing ER stress, successfully prevents TM cell death and lowers IOP in glaucoma models associated with MYOC mutations [142]. In addition, mutant myocilin proteins interact with components of the extracellular matrix (ECM), including fibronectin, elastin, and collagen type IV and I, resulting in aberrant accumulation of ECM proteins in the ER and dysregulation of the ECM, which contributes to reduced outflow of aqueous humor and increased IOP in some glaucoma cases [144]. Inhibition of PERK by GSK2606414 reduces cell survival, while activation of this pathway by salubrinal, which inhibits elF2α dephosphorylation, increases cell survival, suggesting a protective effect of PERK activation in stressed TM cells [145]. Interestingly, in another study, inhibition of PERK by LDN-0060609 was shown to reduce DNA damage, improve cell survival and restore cell function in human TM cells [146]. The paradoxical results from the two studies may be in part attributable to the specific pharmacological inhibitors or stress conditions; further investigation of these compounds and which downstream pathways they affect is essential for the development of therapies that incorporate them.

**Fig. 2:**
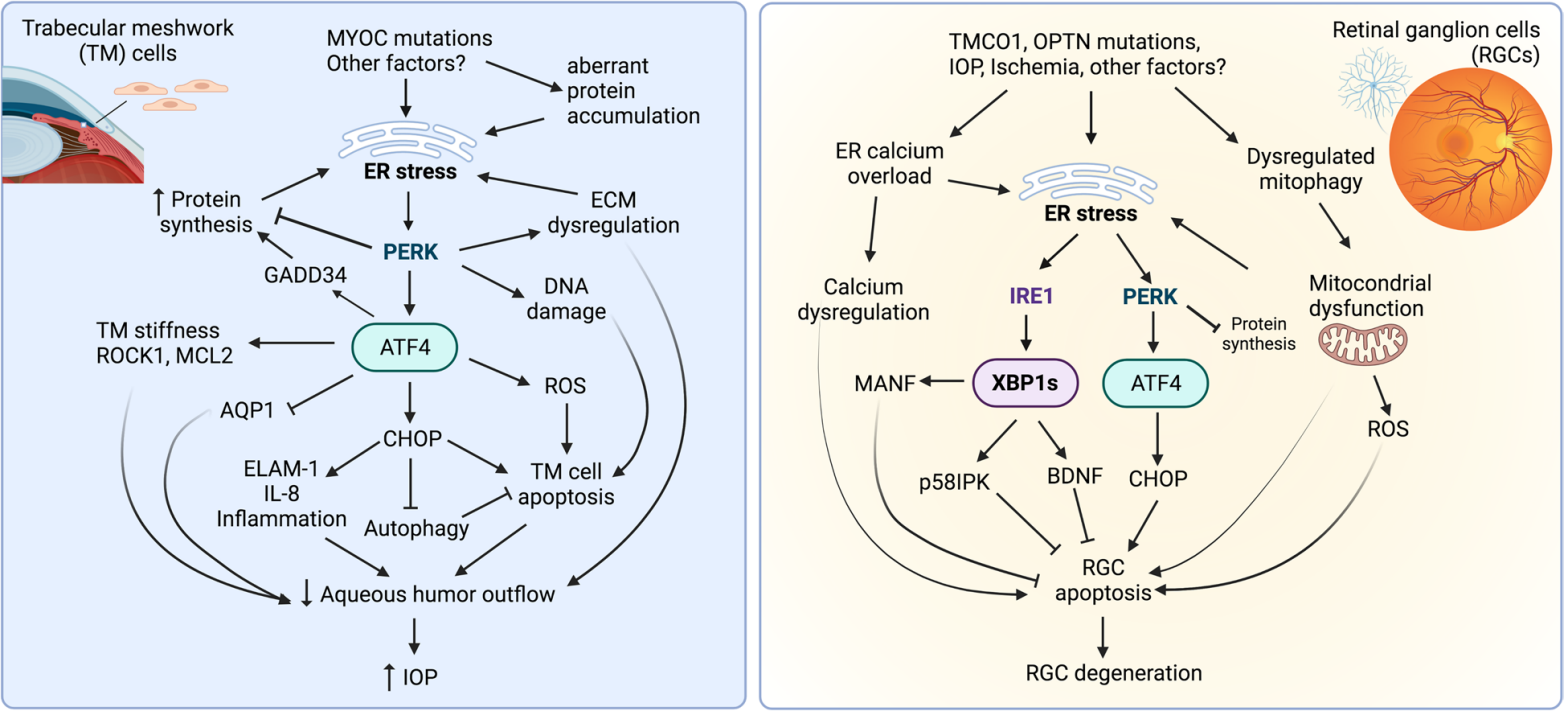
Implication of the UPR pathways in the pathogenesis of glaucoma. Left panel: Mutations of MYOC gene and other factors induces ER stress resulting in activation of the PERK/ATF4/CHOP pathway. Activation of this pathway leads to increased reactive oxygen species (ROS), inflammation, and DNA damage, promoting TM cell apoptosis. In addition, ATF4 increases TM stiffness contributing to reduced outflow of aqueous humor and increased IOP. Right panel: ER stress is induced by multiple factors, including mutations of genes such as TMCO1 and OPTN, increased IOP, ischemia, and others, in retinal ganglion cells (RGCs) during glaucoma. Activation of XBP1 increases expressions of ER chaperones and neurotrophic factors, such as brain derived neurotrophic factor (BDNF), p58^IPK^, and mesencephalic astrocyte-derived neurotrophic factor (MANF), reducing apoptosis of RGCs. Activation of ATF4/CHOP, mitochondrial dysfunction, and calcium dyshomeostasis, contribute to RGC cell death and degeneration

ATF4 is a major downstream effector in the PERK pathway and studying this component of the pathway can help to better understand the conflicting evidence previously discussed on PERK. In this mechanism, elF2α phosphorylation increases ATF4 protein production while reducing global protein translation. As a transcription factor, ATF4 binds to the promotor of the aquaporin 1 (AQP1) gene and negatively regulates its transcription in TM cells [146, 147]. Reduced expression of AQP1 is believed to be responsible for increased resistance to aqueous humor outflow that leads to elevated IOP in glaucoma associated with increased endothelin-1 (ET-1) level in aqueous humor [147]. In addition, activation of the elF2α/ATF4/CHOP pathway increases apoptosis and inflammation in human TM cells, in part through promoting ER stress-induced apoptosis, increasing ROS production, upregulating inflammatory genes such as endothelial-leukocyte adhesion molecule 1 and Interleukin 8 [148]. Activation of ATF4 also results in increased protein synthesis that increases the ER protein load, thereby exacerbating ER stress in TM cells [149]. Importantly, increased ATF4 and CHOP expression have been observed in TM from patients with POAG, suggesting that the activation of ATF4/CHOP pathway is implicated in TM cell injury and IOP increase in human glaucoma [148–150]. In addition to primary glaucoma, elevated ER stress in TM cells has been implicated in dexamethasone-induced ocular hypertension, which resembles glucocorticoid-induced glaucoma in human patients [151]. Relative to the ATF4/CHOP pathway, the implication of the IRE1/XBP1 and ATF6 UPR branches in ER stress-associated TM cell dysfunction and cell death are less well studied (Fig. 2). Future studies are warranted to investigate whether targeting these understudied UPR pathways may lead to new avenues for reducing TM injury and inflammation in glaucoma models.

#### Cellular stress signaling in RGC damage

Like in TM cells, ER stress plays a pivotal role of in RGC cell death associated with glaucoma [152–155]. Some examples are RGC injuries caused by genetic variants of transmembrane and coiled-coil domain 1 (TMCO1) and optineurin (OPTN). TMCO1 encodes a transmembrane protein of the ER and functions as a calcium leak channel to prevent calcium overload and maintain calcium homeostasis in the ER [156]. TMCO1 is expressed ubiquitously in the body with high expression in RGCs and a genetic variant was recently identified as a risk factor for POAG [157, 158]. Deficiency or dysfunction of TMCO1 induces calcium overload in the ER, which in turn causes disturbance in protein synthesis and folding resulting in ER stress. Dysregulation of calcium signaling also increases ROS generation, over-activates mitophagy resulting in mitochondrial damage and impaired respiratory function, and promotes apoptosis [157, 159, 160]. OPTN encodes a protein that functions as a primary receptor of mitophagy and multiple mutations of OPTN protein have been identified associated with POAG [161]. Among these mutations, E50K is considered the most prevalent and is associated with normal-tension glaucoma, a subtype of POAG [162]. Overexpression of E50K mutant optineurin induces mitochondrial fission and enhanced mitochondrial degradation and mitophagy resulting in RGC degeneration [162]. Dysregulation of mitochondrial fission and mitophagy increases oxidative stress, which further intensifies mitochondrial dysfunction and damage resulting in a vicious cycle ultimately contributing to RGC cell death [163]. Another glaucoma-associated mutation of OPTN, 691_692insAG (or 2bpIns-OPTN), was shown to increase ER stress and upregulate CHOP expression resulting in cell death [164]. Future studies should investigate whether inhibition of ER stress prevents RGC degeneration induced by OPTN mutations in animal models of glaucoma.

AMPK is an energy sensor and a master regulator of cellular metabolism and mitochondrial dynamics [34]. However, the role of AMPK in regulation of energy homeostasis and mitochondrial function in RGCs and glaucoma appears to be less thoroughly investigated. A recent study demonstrates that AMPK is activated in RGCs in an ocular hypertension mouse model and in human glaucomatous retina tissue from patients with POAG [69]. Sustained activation of AMPK triggers RGC dysfunction and leads to RGC dendritic retraction and synapse elimination through inhibiting mammalian target of rapamycin complex 1 (mTORC1). Furthermore, when AMPK is depleted, RGC survival and retinal function is improved. These results suggest that chronic AMPK activation contributes to RGC cell death perhaps by inhibiting the energy consuming processes such as synaptic transmission and axon transport [69]. This finding is in apparent contrast to the protective role of AMPK in AMD (as described above) in which activation of AMPK mitigates photoreceptor and RPE degeneration. These discrepancies highlight the importance in understanding the signaling pathways in each specific type of neurons, which may possess unique mechanisms to combat different stresses and disease conditions.

In addition to metabolic disturbance, ER stress has been observed in RGCs in several animal models of glaucoma, including microbeads-induced ocular hypertension model, optic nerve crush model, and DBA/2 J (D2) mouse model [165–167]. Activation of the UPR pathways appears to play differential roles in glaucomatous RGC damage. Activation of the IRE1/XBP1 pathway protects RGCs from ER stress-induced damage in part through increasing expression of brain derived neurotrophic factor (BDNF); conversely, activation of the PERK-eIF2α-CHOP pathway can trigger RGC apoptosis [167, 168]. Combining the two approaches of over-expression of XBP1 and inhibition of eIF2α phosphorylation has been shown to not only protect RGC survival but also protect against axon degeneration and improve visual function in mouse models of traumatic optic nerve injury and microbeads-induced ocular hypertension [166]. However, in DBA/2 J mice deletion of CHOP results in modest protection to the RGC soma but does not protect against RGC axonal degeneration [165]. This could suggest that additional downstream effectors in the PERK/eIF2α pathway could be involved in RGC injury related to glaucoma.

Recent work demonstrates a potential role of an ER-resident chaperone p58^IPK^ in RGC survival in glaucomatous conditions [169–171]. p58^IPK^ is a multifunctional protein that acts as a co-chaperone of GRP78 in the process of protein folding and also plays a role in regulation of eIF2α phosphorylation, and thereby protein production, by inhibiting eIF2α kinases including double-stranded RNA-dependent protein kinase R [172–176], PERK [177, 178], and GCN2 (general control nonderepressible 2) [179]. p58^IPK^ is highly expressed in the neural retina and its expression is upregulated under ER stress conditions [169]. Deletion of p58^IPK^ results in fewer RGCs, accompanied by increased levels of CHOP and Bax (Bcl-2 Associated X-protein) in the retina of p58^IPK^ knockout (KO) mice, and moreover, the p58^IPK^ KOs are highly susceptible to ischemia-induced RGC loss compared to the wild-type animals. Conversely, overexpression of p58^IPK^ attenuates oxidative stress and ER stress-induced apoptosis of cultured neural cells, suggesting a protective role of p58^IPK^ in retinal neurons [169]. Overexpressing p58^IPK^ using AAV protects against ER stress-induced cell death in cultured primary RGCs from both WT and p58^IPK^ knockout mice [171]. In addition to p58^IPK^, recent studies identified mesencephalic astrocyte-derived neurotrophic factor (MANF) as an ER-localized neurotrophic factor, which inhibits ER stress-induced cell death of retinal neurons and improves RGC survival in a rat glaucoma model [171]. Therefore, enhancing the function of ER chaperones like p58^IPK^ and MANF to restore protein homeostasis may offer exciting therapeutic potential for glaucomatous RGC degeneration (Fig. 2).

### Diabetic retinopathy

Diabetic retinopathy (DR) is a major complication of diabetes characterized by progressive neurovascular injury and degeneration in the retina and is the most frequent cause of blindness in working-age adults. According to clinical manifestations, DR is classified into two large categories: non-proliferative DR (NPDR) and proliferative DR (PDR), representing the early and advanced stages of the disease, respectively. Major pathological characterization of NPDR includes retinal hemorrhages, microaneurysms, microvascular abnormalities, while PDR is distinguished by the development of retinal neovascularization (NV) due to aberrant blood vessel growth from the retina into the vitreous [6, 180, 181]. The fragile and malstructured blood vessels of retinal NV are prone to leakage and rupture, resulting in severe vitreous hemorrhage, fibrosis, tractional retinal detachment, and vision loss [180–182]. Leakage of injured retinal blood vessels and disruption of the BRB can also occur at early stages of DR, resulting in exudates and fluid accumulation in retinal tissue and thickening of the retina, known as diabetic macular edema (DME). DME is the most frequent cause of central vision loss in diabetic patients. In addition to vascular lesions, recent work recognizes the importance of diabetes-induced neural retina dysfunction and neurodegeneration in DR, although effective treatment for protection of retinal neurons and prevention of vision loss in DR is not yet available [183–187]. Currently, clinical managements for DR focus primarily on reducing vascular pathologies using a combination of anti-VEGF therapy, laser photocoagulation, and surgical treatment [188]. In many patients, in particular those with advanced DR, successful treatment in correcting vascular abnormalities and restoring the anatomical structure of the retina does not result in significant visual improvement [189]. New approaches to protect retinal cells and improve retinal function are urgently needed.

A number of molecular pathways and cellular processes, such as oxidative stress, ER stress, and inflammation, have been proposed in DR pathogenesis. Oxidative stress is considered a primary cause of retinal vascular damage in diabetes [190]. Major pathways contributing to ROS generation in diabetic retinal cells include activation of polyol and hexosamine biosynthetic pathways, advanced glycation end product (AGEs) production, protein kinase C (PKC) activation, mitochondrial dysfunction, and NADPH (nicotinamide adenine dinucleotide phosphate) oxidase activation [181, 191]. In addition, defects in the anti-oxidant defenses that scavenge free radicals and reduce oxidative stress also contribute to oxidative damage in the diabetic retina [192]. Studies have shown that during diabetes the DNA binding ability of Nrf2 is significantly reduced in retinal cells, and in contrast, the binding between Nrf2 and its inhibitor, Kelch like-ECH-associated protein 1 (Keap1) is increased resulting in enhanced Nrf2 degradation and decreased Nrf2 translocation to the nucleus [193, 194]. Inhibition of Keap1-Nrf2 interaction by small molecules to promote Nrf2 nuclear translocation and transcription activation of anti-oxidant defense genes alleviates oxidative stress, protects retinal cells from ischemic and inflammatory injury, and mitigates diabetic vascular damage [193, 195]. These findings suggest that targeting the anti-oxidant defense system and enhancing the cellular response to dampen oxidative stress and minimize oxidative damage of retinal cells could be a promising strategy for prevention and treatment of early-stage DR.

**Fig. 3:**
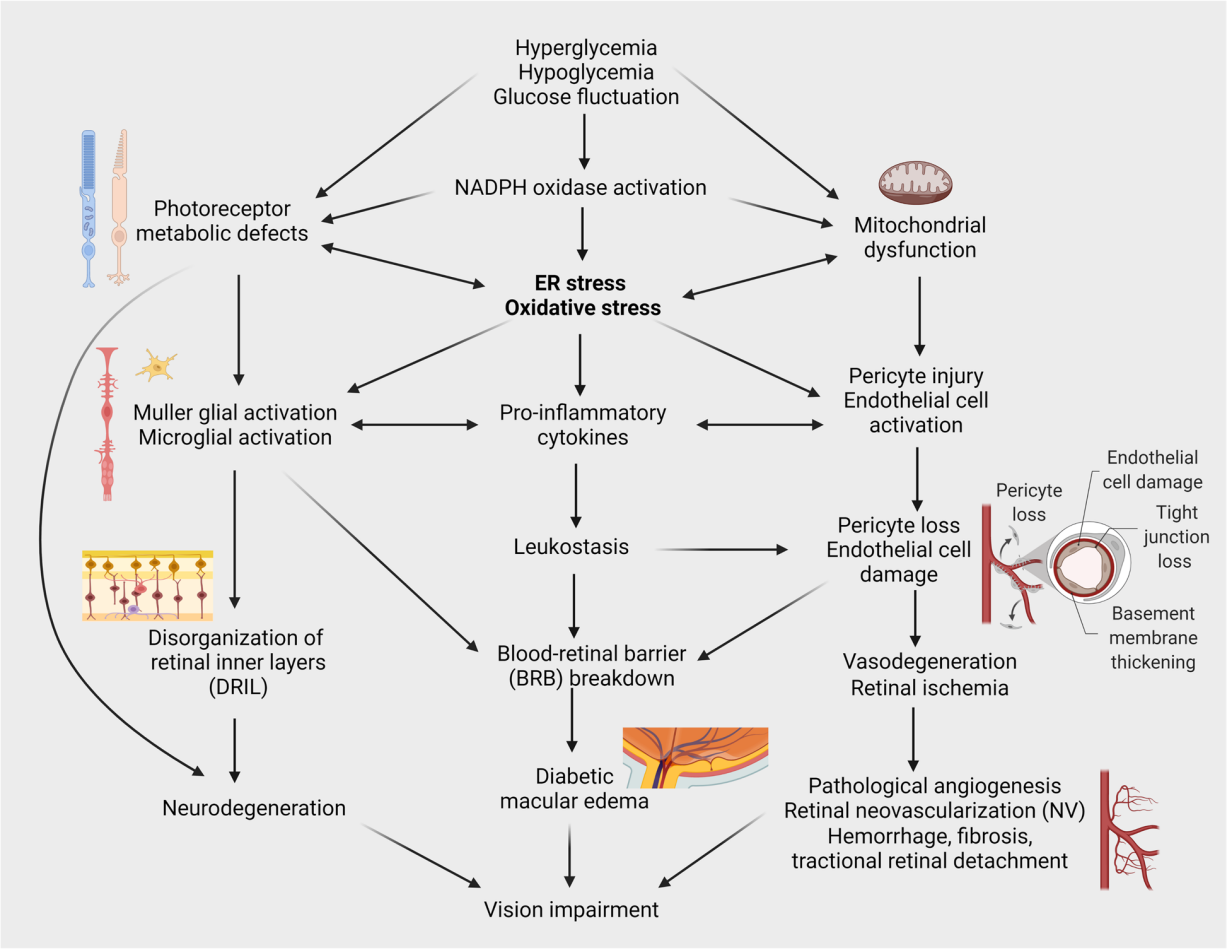
Role of ER stress and oxidative stress in retinal neurovascular damage in diabetic retinopathy (DR). Disturbance in glucose supply (hyperglycemia, hypoglycemia, glucose fluctuation, etc.) leads to metabolic defects, mitochondrial dysfunction, NADPH oxidase activation, resulting in increased ER stress and oxidative stress. Enhanced ER stress and oxidative stress play a central role in inducing vascular endothelial cell and pericyte damage, blood-retinal barrier (BRB) breakdown, glial activation, inflammation and diabetic macular edema. Cumulative loss of vascular cells and capillaries resulting in retinal ischemia, which ultimately leads to retinal neovascularization and neurodegeneration contributing to vision impairment in DR

In addition to oxidative stress, ER stress has been shown to play a significant role in diabetes-associated retinal inflammation, endothelial cell injury, vascular leakage and vascular degeneration (Fig. 3) [196–202]. Recent studies also highlight the importance of the UPR signaling in maintaining retinal neuronal function and preventing neurodegeneration in diabetic conditions [203, 204]. Activation of the IRE1/XBP1 and PERK/ATF4/CHOP pathways differentially regulate retinal endothelial cell death, inflammation, and vascular permeability in animal models of diabetes [196, 199, 200, 205–207]. Preconditioning with mild ER stress activates XBP1-dependent UPR pathways, reducing retinal endothelial inflammation and vascular leakage [197]. Conversely, loss of XBP1 induces Müller glia activation and promotes retinal inflammation in DR [208]. Moreover, cells deficient of XBP1 are susceptible to oxidative stress-induced apoptosis and cell death and tight junction damage [74, 76, 79, 80]. These findings imply a vital role of XBP1 in maintaining cellular function and integrity in diabetic retinas. Conditional knockout of XBP1 in retinal neurons leads to early onset retinal function decline, neuronal loss, and enhanced Müller glia activation in diabetic mice [203], suggesting that the XBP1 pathway is critical for neuronal protection against diabetes induced retinal injury and dysfunction.

The retina has high metabolic demands to support its function in generating and transmitting visual signals and maintain the normal structure of photoreceptors. In diabetes, retinal metabolism is disrupted due to elevated glucose levels, correlated with enhanced glycolysis and sorbitol oxidation, which has been implicated in the pathogenesis of DR [209–211]. Diverting upstream metabolites from glycolysis into other pathways, such as the hexosamine, diacylglycerol (DAG)/PKC, and AGE pathways, leads to endothelial injury in diabetes [212]. Systemic reduction of GLUT1 or deletion of GLUT1 in retinal neurons prevents polyol accumulation and improves retinal function in diabetic animals, suggesting a role of metabolic dysregulation in neurodegeneration in DR [209]. In addition, mitochondrial dysfunction and damage leads to reduced mitochondrial respiratory activity further contributing to the imbalance between glycolysis and oxidative phosphorylation in diabetic retinal cells [reviewed in [213]. As a major cellular stress response, the UPR has been shown to play an important role in regulation of glucose metabolism in retinal cells [18, 214]. The IRE1 branch functions as a nutrition sensor in cells under starvation and induces activation of XBP1 to restore energy homeostasis [215]. In glioma cells, silencing XBP1 suppresses hexokinase-2 (HK2) therefore inhibiting glycolysis and resulting in cell death [216]. Conditional knockout of XBP1 in retinal cells also leads to reduced glycolysis associated with retinal dysfunction and neurodegeneration [18], suggesting a role of XBP1 in regulation of retinal neuronal glycolysis. The exact function of XBP1 and other UPR pathways in regulation of retinal metabolism during diabetes remains to be elucidated.

## Conclusions

The long-term and constant requirement for the retina to maintain protein and metabolic homeostasis is critical for preserving normal visual function and preventing retinal neurodegeneration throughout the lifetime. Studies over the past two decades have laid a groundwork for understanding how elements of the UPR respond to various stressors during aging and in common retinal disease conditions including AMD, RP, glaucoma, and DR in humans and in animal models. The findings reported so far clearly suggest that activation of the UPR signaling has a significant impact on retinal cell survival and function, not only through governing the homeostasis of protein production, modification, trafficking, and degradation, but also via regulation of cell metabolism, mitochondrial function, and calcium levels. Although the interactions between the UPR pathways, as well as their involvement in metabolic regulation, can vary in different cell types and are not necessarily consistent between disease conditions, the work described in this review provides hope that targeting the UPR pathways may lead to new therapeutic approaches for protecting retinal cells at the early stages of neurodegenerative disease.

As discussed earlier, aging is a significant risk factor for major neurodegenerative diseases in the retina, as it is for Alzheimer’s disease, Parkinson’s disease, and many others in the CNS. Aberrant protein aggregation and deposition, along with enhanced protein and lipid oxidation, correlate with chronic ER stress and oxidative stress in aging retinal tissue [18, 30, 217, 218]. While the disruption of proteostasis can be attributable to declined ability to activate the protective UPR pathways in aged cells [18], the mechanisms behind the dysfunction of the UPR during aging remain poorly understood. Several factors have been proposed to potentially mediate the failure of sensing ER stress and activation of the UPR, including disturbed redox balance in the ER, dysregulated calcium homeostasis, and increased nitrosylation of ER stress sensors and ER chaperones or foldases [219]. Whether targeting these factors could restore the function of the UPR in aging and diseased retinal cells warrants future investigation.

Another interesting question is how the UPR pathways interact and reciprocally regulate metabolic signaling pathways in retinal cells. The role of the UPR in metabolic diseases including obesity and diabetes has been extensively investigated. In addition to restoring the ER and protein homeostasis thereby improving cell survival and function, the UPR genes have also been shown to independently regulate pathways in glucose and lipid metabolism. Furthermore, multiple UPR molecules directly and indirectly regulate critical genes responsible for anti-oxidant defense and mitochondrial function. Yet the exact mechanisms by which the UPR signaling is implicated in metabolic regulation in response to stressors in each disease condition and in various retinal cell types are largely unknown. Understanding the interactions between these signaling pathways in coordinating cellular stress responses to maintain and improve the capacity for metabolic regulation and protein homeostasis could provide valuable insight for therapeutic intervention.

It is important to recognize that the retina is capable of dealing with significant cellular stress on a daily basis, often for decades, without significant functional decline or neurodegeneration even under disease conditions. The concept that an additional cause, such as compromised nutrient sensing due to advanced age or the breakdown of the BRB, is required for cellular stress response pathways to be overwhelmed thereby leading to functional decline and neurodegeneration is particularly intriguing. Recent development of new technologies, such as single cell multi-omics that enable multiple, and even simultaneous, genetic, transcriptomic, epigenetic, and proteomic analyses from individual cells using tissue sections [220], could generate precise information on the temporal and spatial changes of each signaling molecule in the UPR pathways in the retina during aging and under disease conditions. Furthermore, the emerging new experimental systems, including stem cell-derived human organoids and humanized animal models, demonstrate remarkable advantage in studying human retinal development and diseases [221]. Last but not least, the successful discovery of small molecules and pharmacological compounds targeting selective UPR signaling (reviewed in [108]) provides valuable tools for better understanding the implication of individual UPR pathways in disease progression and opens new avenues for developing drug treatments for retinal protection against neurodegeneration.

## Data Availability

Not applicable.

## Abbreviations

AAV: Adeno-associated virus
AGE: Advanced glycation end product
AMD: Age-related macular degeneration
AMPKα1: AMP activated protein kinase, alpha 1
AMPKα2: AMP activated protein kinase, alpha 2
APP: Amyloid precursor protein
AQP1: Aquaporin 1
ARMS2: Age-related maculopathy susceptibility 2
ATF4: Activating transcription factor 4
ATF6: Activating transcription factor 6
Bax: Bcl-2 Associated X-protein
BDNF: Brain derived neurotrophic factor
BiP: Immunoglobulin binding protein
BRB: Blood-retinal barrier
CAV1: Caveolin-1
CAV2: Caveolin-2
CHOP: C/EBP homologous protein
CFH: Complement factor H
cKO: Conditional knockout
CNS: Central nervous system
CNV: Choroidal neovascularization
DHA: di-docosahexaenoic acid
DME: Diabetic macular edema
DR: Diabetic retinopathy
ECM: Extracellular matrix
eIF2α: Eukaryotic translation initiation factor-2α
Emc3: ER membrane protein complex 3
ER: Endoplasmic reticulum
ERAD: ER-associated degradation
ERG: Electroretinogram
ET-1: Endothelin-1
GA: Geographic atrophy
GAS7: Growth arrest-specific protein 7
GCN2: General control nonderepressible 2
GLUT1: Glucose transporter 1
GRP78: Glucose-regulated protein 78
HK2: Hexokinase-2
HRD1: HMG-CoA reductase degradation protein 1
IOP: Intraocular pressure
IRE1: Inositol requiring enzyme 1
ISR: Integrated stress response
JOAG: Juvenile open-angle glaucoma
Keap1: Kelch like-ECH-associated protein 1
Lkb1: Liver kinase B1
MANF: Mesencephalic astrocyte-derived neurotrophic factor
MNV: Macular neovascularization
mTOR: Mammalian target of rapamycin
mTORC1: mTOR complex 1
MYOC: Myocilin
NADPH: Nicotinamide adenine dinucleotide phosphate
nAMD: Neovascular AMD
NPDR: Non-proliferative DR
Nrf2: NF-E2-related factor 2
NV: Neovascularization
OCT: Optical coherence tomography
ONL: Outer nuclear layer
OPTN: Optineurin
OS: Outer segments
p58^IPK^: 58 kDa inhibitor protein kinase
PACG: Primary angle-closure glaucoma
PAX6: Paired Box Gene 6
PBA: Phenylbutyric acid
PDR: Proliferative DR
PERK: PKR-like endoplasmic reticulum kinase
PKC: Protein kinase C
POAG: Primary open-angle glaucoma
POMC: Pro-opio-melanocortin
POS: Photoreceptor outer segments
RGCs: Retinal ganglion cells
RHO: Rhodopsin gene
RIDD: Regulated IRE1-dependent mRNA decay
ROS: Reactive oxygen species
RP: Retinitis pigmentosa
RPE: Retinal pigment epithelium
TM: Trabecular meshwork
TMCO1: Transmembrane and coiled-coil domain 1
UPR: Unfolded protein response
VEGF: Vascular endothelial growth factor
WT: Wild type
XBP1: X-Box Binding Protein 1
XBP1s: Spliced XBP1
